# Eyeblink Conditioning and Novel Object Recognition in the Rabbit: Behavioral Paradigms for Assaying Psychiatric Diseases

**DOI:** 10.3389/fpsyt.2015.00142

**Published:** 2015-10-07

**Authors:** Craig Weiss, John F. Disterhoft

**Affiliations:** ^1^Department of Physiology, Northwestern University Feinberg School of Medicine, Chicago, IL, USA

**Keywords:** Alzheimer’s disease, cerebellum, cognitive dysmetria, hippocampus, prefrontal cortex, schizophrenia

## Abstract

Analysis of data collected from behavioral paradigms has provided important information for understanding the etiology and progression of diseases that involve neural regions mediating abnormal behavior. The trace eyeblink conditioning (EBC) paradigm is particularly suited to examine cerebro-cerebellar interactions since the paradigm requires the cerebellum, forebrain, and awareness of the stimulus contingencies. Impairments in acquiring EBC have been noted in several neuropsychiatric conditions, including schizophrenia, Alzheimer’s disease (AD), progressive supranuclear palsy, and post-traumatic stress disorder. Although several species have been used to examine EBC, the rabbit is unique in its tolerance for restraint, which facilitates imaging, its relatively large skull that facilitates chronic neuronal recordings, a genetic sequence for amyloid that is identical to humans which makes it a valuable model to study AD, and in contrast to rodents, it has a striatum that is differentiated into a caudate and a putamen that facilitates analysis of diseases involving the striatum. This review focuses on EBC during schizophrenia and AD since impairments in cerebro-cerebellar connections have been hypothesized to lead to a cognitive dysmetria. We also relate EBC to conditioned avoidance responses that are more often examined for effects of antipsychotic medications, and we propose that an analysis of novel object recognition (NOR) may add to our understanding of how the underlying neural circuitry has changed during disease states. We propose that the EBC and NOR paradigms will help to determine which therapeutics are effective for treating the cognitive aspects of schizophrenia and AD, and that neuroimaging may reveal biomarkers of the diseases and help to evaluate potential therapeutics. The rabbit, thus, provides an important translational system for studying neural mechanisms mediating maladaptive behaviors that underlie some psychiatric diseases, especially cognitive impairments associated with schizophrenia and AD, and object recognition provides a simple test of memory that can corroborate the results of EBC.

Neuropsychiatric diseases are a significant worldwide health issue. Analysis of data collected from behavioral paradigms has provided important information for understanding the etiology, and progression of diseases that involve neural regions mediating abnormal behavior. Behavioral paradigms also provide systems for testing potential treatments and therapeutics. Eyeblink conditioning (EBC) is one such behavioral paradigm. This paradigm pairs a neutral conditioning stimulus (CS), e.g., a brief tone, flash of light, or vibration of whiskers with a mildly aversive stimulus to the eye or surrounding area in order to evoke a conditioned blink response. Subjects become conditioned after several pairings of the stimuli such that a blink is evoked in response to the CS and prior to the onset of the aversive unconditioned stimulus (US). Importantly, control experiments indicate that the learning is associative in nature, i.e., blinks do not tend to occur to the CS when it is presented in a random unpaired schedule with the US.

Learning occurs most quickly when onset of the US is delayed from the onset of the CS by approximately 250 ms, and when the CS and US overlap and coterminate in time [longer interstimulus intervals (ISIs) are optimal for human subjects]. The 250 ms ISI is the shortest interval tested in the rabbit by Schneiderman and Gormezano (1). Several studies have found that generation of a conditioned response (CR), a blink that occurs prior to the onset of the US and which protects the eye from the noxious stimulus, requires the thalamus, cerebellum, and afferent inputs from the brainstem to the cerebellum (2–5). However, learning the task is more difficult when a stimulus-free interval separates the two stimuli during a trial, i.e., more trials are required before CRs are exhibited (6). The simple addition of this stimulus-free “trace” interval between the two stimuli increases the memory demand of the task, recruits forebrain areas that would otherwise not be required for the task, and importantly requires awareness that the CS predicts the occurrence of the aversive stimulus [as reported by human subjects (7, 8)]. The requirement for awareness makes trace EBC a useful paradigm to investigate the cognitive nature of cerebellar function as proposed by Leiner et al. (9, 10), and abnormalities in the cerebro-cerebellar circuitry that mediates awareness likely involves the circuitry that makes EBC sensitive to neuropsychiatric disease.

The distinction between the neural requirements for the delay and trace versions of the EBC paradigm allows behavioral testing to dissociate forebrain-dependent cognitive effects from a more basic sensorimotor integration mediated by the brainstem/cerebellar/thalamic systems. Although EBC has been used most often to study neural mechanisms mediating learning and memory in healthy adults, the dissociation between forebrain and cerebellar/brainstem effects is useful in helping to characterize the effects of a disease state, and the effects of a potential treatment.

Several reports indicate that EBC can be used to detect impairments in neuropsychiatric diseases, such as schizophrenia (11–14), Alzheimer’s disease [AD (15–17)], progressive supranuclear palsy [PSP (18)], and post-traumatic stress disorder [PTSD (19)] EBC is significantly impaired by AD, relative to age-matched control subjects (15, 17). There is the one report of EBC in patients with PSP, which indicates a severe impairment in acquiring EBC with trace intervals of 0, 300, or 600 ms (18); those authors concluded that the deficit was likely due to neuropathological changes in the cerebellar nuclei since other pathologies overlap with those of Parkinson’s disease (PD), which does not impair acquisition of EBC (20). The effects of PTSD on EBC are discussed by Schreurs and Burhans elsewhere in this volume (19). The EBC paradigm also reveals age-related learning impairments in humans (21–23), rabbits (24), and rats (25–28). Overall, the EBC paradigm is quite translational in nature. The phases of behavioral acquisition are similar between human and non-human subjects (although scaled differently) and many of the same stimuli and stimulus delivery systems can be used with both types of subjects (29). Much of our understanding of the neural networks mediating this conditioning comes from *in vivo* recordings from single neurons and multiunit activity in different brain regions during the task (30–35), and from permanent and temporary lesions of regions suspected to be involved in the task (3, 4, 33, 36–39).

Although this review focuses on the benefits of using the rabbit as the experimental subject, considerable advances have been made by using the mouse as a subject for EBC and deserve mention, especially for manipulations of the cerebellum and different transmitter systems. An understanding of the neurotransmitters and receptors involved in conditioning and cognition has been facilitated by using knockout and transgenic mice, e.g., elimination of monoamine oxidase isoenzymes A and B increases levels of monoamines, including serotonin (40) and resulted in abnormally enhanced acquisition rates of delay EBC, elevated levels of hippocampal long-term potentiation, decreased ratio levels of NMDA receptor subunits NR2A and NR2B in prefrontal cortex (PFC) [increased ratio levels in hippocampus (41)] and the adenosine receptor has been shown to be important in both acquisition of EBC and the development of LTP (42). These studies are of interest given the involvement of NMDA receptors and serotonin in schizophrenia (43–45).

In terms of the cerebellum, elimination of cannabinoid receptor 1 (CB1), which is highly expressed in cerebellum, or mutations of the glutamate receptor mGluR1 (46) or subunit delta2 which affects cerebellar cortex was found to significantly impair delay conditioning, but not trace conditioning [(47, 48), see Ref. (49) for a discussion of this result], and elimination of calcium/calmodulin-dependent protein kinase type IV (CaMKIV), which is expressed in cerebellar granule and nuclear cells, impaired long-term retention of delay conditioned blinks (50). These studies are of interest given the role of cerebellar–cortical interactions with schizophrenia (51).

In terms of AD, the insertion of genes related to AD have been shown to accelerate impairments in mice acquiring EBC (52, 53) and reduce the volume of their hippocampus, as measured with MRI (54). However, the genetic sequence for amyloid in the mouse is different than the sequence found in human amyloid. This adds the complication of foreign DNA in the host. By contrast, the rabbit sequence for amyloid is identical to the sequence in humans (55) and should minimize that complication. Lastly, learning specific changes in the cortical representation of the CS for whisker-signaled conditioning have been described (56) and provide a substrate for experimental manipulation.

A circuit diagram of relevant brain regions involved in trace and delay EBC is shown in Figure 1. Note that five modules have been identified: cerebellum, PFC, limbic-medial temporal, sensory cortex, and basal ganglia. The thalamic nuclei connecting the different modules are also shown (the anterior thalamus (AT) includes anterior dorsal, anterior ventral, and anterior medial). The circuit shows the flow of information representing the conditioning stimuli through the cerebellum, the forebrain, and back to the cerebellum by way of the pontine nuclei. The disruption of any of the pathways or nuclei will lead to maladaptive responses to the stimuli regulating learned behaviors and to disrupted executive functions due to changes in the PFC.

**Figure 1:**
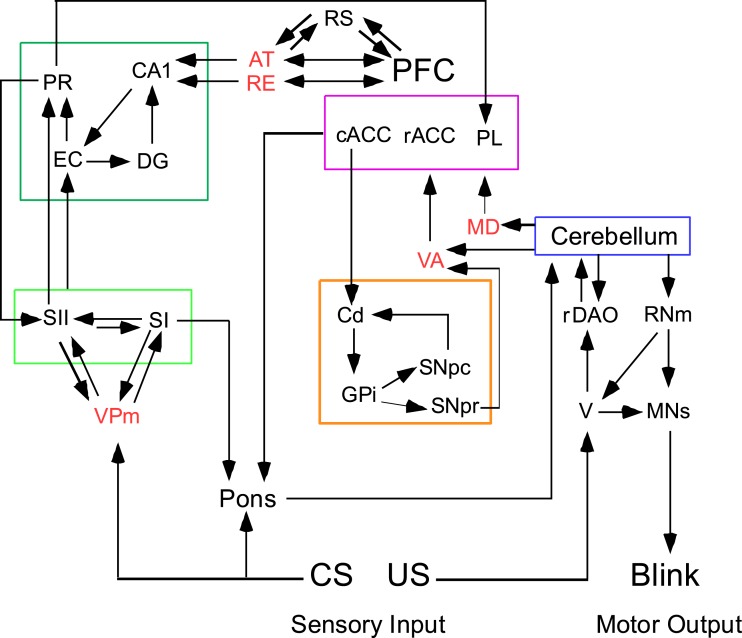
**Trace eyeblink conditioning requires forebrain input to a cerebellar circuit that mediates conditioning**. Four forebrain circuits that interact via thalamic nuclei (shown in red text). The limbic, medial temporal circuit is outlined in dark green and is sensitive the effects of aging. The limbic forebrain circuit is outlined in magenta and is affected during schizophrenia. The basal ganglia circuit is outlined in tan and is affected during supranuclear palsy. The sensory circuit is outlined in light green (the somatosensory system is shown in this example). The cerebellar circuit is shown to the far right. The conditioning stimulus (CS) is conveyed to the cerebellum via mossy fibers originating in the pontine nuclei; the unconditioned stimulus (US) is conveyed via climbing fibers from the inferior olive. AT, anterior thalamus; cACC, caudal anterior cortex; Cd, caudate; DG, dentate gyrus; EC, entorhinal cortex; GPi, globus pallidus internal; MD, medial dorsal thalamus; MNs, motor neurons (facial and accessory abducens for blink conditioning); PFC, prefrontal cortex; PL, prelimbic cortex; PR, perirhinal cortex; rACC, rostral anterior cingulate cortex; rDAO, rostral dorsal accessory olive; RE, nucleus reuniens; RNm, magnocellular red nucleus; RS, retrosplenial cortex; SI, primary sensory cortex; SII, secondary sensory cortex; V, trigeminal nucleus; VA, ventral anterior thalamus; VPm, ventral posterior medial cortex; SNpc, Substantia Nigra pars compacta; SNpr, Substantia Nigra pars reticulata.

The cerebellum is a necessary component for acquisition and expression of conditioned blink responses (2, 57). It is one synapse removed from the motor neurons that control the CR and importantly, it provides feedback to the frontal cortex via the thalamus (58–60). Removal of this input may contribute to a cognitive dysmetria and symptoms of schizophrenia (51). Destruction of the cerebellar nuclei (the sole output of the cerebellum) eliminates acquisition and expression of CRs, but leaves intact the unconditioned, reflexive eyeblink to noxious stimuli. The PFC is required for acquisition of EBC when the task is cognitively demanding as in trace conditioning or when the CS is relatively mild and requires attention for detection, even during delay conditioning (61). Acquisition of trace EBC requires the caudal anterior cingulate portion of the PFC [cACC (36)], and long-term retention involves the prelimbic (PL) portion (35). Lesions of the hippocampus result in non-adaptive short-latency CRs or with enough damage the animal is unable to acquire CRs (6, 62). Lesions of SI prior to whisker-signaled trace EBC prevent acquisition of CRs, but similar lesions made after consolidation has been allowed to occur for 30 days does not abolish CRs. We suggest that CS information is relayed into the hippocampal formation via the secondary sensory cortical system after consolidation has occurred. The role of the striatum was examined because of cognitive deficits associated with PD (63–66). Lesions of the caudate nucleus prevent acquisition of CRs (33) and similar lesions made after acquisition prevent any further improvement in expression of the CR (67).

Although most recording and lesion techniques are invasive and not appropriate to study in humans, functional magnetic resonance imaging can be done in both human and non-human animal subjects during and after learning (68–70). Blink conditioning thus provides an important translational tool for studying the neural mechanisms mediating maladaptive behaviors that underlie some psychiatric diseases. Here, we review some of the work that has been done with schizophrenia as a prototypical psychiatric disease and suggest ways in which the paradigm may be used to test potential therapeutics.

Other neuropsychiatric diseases have also been examined with EBC, e.g., AD, PSP, PD, and PTSD. Briefly, AD significantly impairs acquisition relative to age-matched control subjects (15), acquisition is normal in patients with PD but impaired in patients with PSP (17, 18), and PTSD has effects (especially on the unconditioned response) as discussed elsewhere in this issue by Schreurs and Burhans (19).

## Schizophrenia

Schizophrenia, a neuropsychiatric syndrome that includes symptoms of hallucinations, delusions, and extremely disordered thinking affects approximately 1% of the population. Behavioral abnormalities related to schizophrenia usually appear in the late teens and causes a life-long disability. Much evidence suggests that schizophrenia is a neuro-developmental disorder affecting connections between the cerebellum and PFC, which leads to a cognitive dysmetria (51, 71). More recently, an analysis of cerebellar gray matter using a modern unbiased morphometry approach, rather than whole-brain voxel based morphometry, found that gray matter volumes in Crus I/II were significantly reduced among patients, and the reduction correlated with tests measuring thought disorders and executive functioning (72).

Schizophrenia should affect both trace and delay conditioning since the cerebellum is required for both the delay and trace versions of the paradigm (73), even though the PFC is not required for the less demanding delay paradigm when salient stimuli are used (36). The connections between the cerebellum and PFC have been studied in non-human primates by Peter Strick and his group (58, 60). They found that neuronal loops connect the dorsolateral PFC and the cerebellum, and that the dentate cerebellar output nucleus of the loop is active during cognitive processing, as measured with functional magnetic resonance imaging [fMRI; (74)]. Cerebellar activation, as measured during fMRI based experiments has yielded mixed results, but a meta-analysis of more than 200 studies (75) found that approximately 40% of reports included individuals with schizophrenia and cerebellar hypoactivation was found in approximately two-thirds of those patients, mostly during tasks testing cognition and executive functions.

We have also used fMRI to measure the blood oxygen level-dependent (BOLD) response from the cerebellum in rabbits conditioned to evoke eyeblinks. We demonstrated learning-related decreases in the cerebellar cortex and learning-related increases in the deep cerebellar nuclei (68). We have also shown with multiple single-neuron tetrode recordings that neurons in the caudal anterior cingulate region (cACC) of the PFC exhibit conditioning specific increases in activity early in the trial sequence that appear to reflect a signal for attention to sensory stimuli. Conversely, neurons in the prelimbic area exhibit robust neuronal activation in response to the CS during tests for retention of remotely acquired EBC, i.e., the rabbits were trained to criterion and then left in their home cages for 30 days (35). Although the exact homolog of the primate dorsolateral PFC is difficult to establish in lower species, the activity pattern we reported for neurons in the prelimbic cortex appears to be a signal that reflects retrieval of the memory for how to respond appropriately to the conditioned stimulus, especially since the activity pattern was not evident during the relatively few trials when CRs were not expressed.

Interactions between the cerebellum and forebrain use relatively long axonal tracts and information processing within the PFC (and elsewhere), and is dependent on the proper functioning of the neurons and interneurons within the region. Abnormalities in GABAergic neurons have been proposed to contribute to the symptoms of schizophrenia. Changes in the inhibitory neurons of the PFC, especially of the dorsolateral PFC, have been reviewed by Lewis et al. (76). They proposed that GABAergic neurons in schizophrenic patients have defects in signaling pathways such that expression of the messenger RNA for GAD67, an enzyme involved in the synthesis of GABA, is reduced and postsynaptic GABA_A_ receptors are upregulated. These deficits in the PFC could account for the disturbances in working memory (43), possibly due to a hypoglutamatergic state since antagonists of NMDA receptors, e.g., ketamine or phencyclidine (PCP), induce hallucinations similar to those observed in people with schizophrenia, and administration of PCP prevents acquisition of trace, but not delay EBC in rabbits (77).

Myelination defects in the cerebellar–prefrontal tracts are also thought to be involved in schizophrenia and have been hypothesized to lead to a functional disconnection between the two regions and a cognitive dysmetria (71). This disconnection could account for the hypoactivation found in the PFC of schizophrenic patients during imaging studies (78). A study of intrinsic connectivity between the cerebellum and the rest of the brain in schizophrenic patients, their siblings, and controls supports the hypothesis of a functional disconnection (79). This study found that patients had significantly impaired connectivity between the cerebellum and forebrain regions, including the hippocampus, thalamus, and middle cingulate gyrus (79). Each of these brain regions, and the cerebellum, are critically involved in mediating trace EBC (3, 4, 6, 31, 36, 38, 79–81).

## Schizophrenia and Blink Conditioning Studies

The literature discussed so far suggest that patients with schizophrenia should have impaired acquisition of both delay and trace EBC because of defects in the cerebellum and thalamus/PFC, respectively. However, initial studies of EBC in patients with schizophrenia yielded mixed results. A review by Lubow (82) concluded that the inconsistencies in results were likely due to differences in the medication history of the patients. Lubow’s conclusion was that the comparison between controls and patients that have or have not been medicated needs to be done in the same study to determine if symptoms are due to the disease *per se*, or due to interactions with medications. Those types of studies have been done (with delay conditioning) since the review by Lubow (11, 13, 82, 83); all of these more recent studies found that the groups with schizophrenia had impaired performance as compared to matched control subjects. The report by Coesmans et al. (83) is noteworthy in that the patients were recently diagnosed with schizophrenia (which limited the effects of medication), and no consistent effect of medication was found on conditioning (clozapine vs. haloperidol), i.e., all patient groups were impaired relative to control subjects. A report by Bolbecker (84) is also noteworthy in that a cerebellar dependence was demonstrated by the subcutaneous administration of secretin (an agonist of group B G-protein coupled receptors), which acts as a retrograde messenger and neuromodulator on cerebellar basket and Purkinje cells. The compound significantly improved delay EBC in medically stable schizophrenic patients, as compared to patients that received a placebo control (controls showed no significant improvement in performance across trial blocks). These data suggest that it is also necessary for the cerebellar cortex to function properly in order for conditioning to occur properly.

Although early studies examining conditioning in schizophrenic patients are difficult to interpret due to differences in medication history, two of the studies are of particular interest in that they measured the level of arousal during the conditioning session. Mednick (85) found that the percentage of CRs correlated with the subjects’ skin potentials, which indicated that the subjects were more aroused. Spain (86) found a similar result, although that experiment may have been confounded by an instruction to press a response key at the termination of the CS (1000 ms CS, 500 ms ISI, 160 ms US). These results are of interest due to interactions with executive functions of the PFC and the sensitivity of the PFC to the modality of the US used during trace conditioning studies. Oswald et al. (87) found that lesions of the PFC [anterior cingulate region (24)] impaired acquisition much more when the US was a puff of air to the cornea as compared to a shock to the periorbital region. The shock US appears to be able to compensate for deficits that might otherwise occur when a less salient stimulus is used.

The effects of arousal on responses to stimuli may be mediated by interactions of the PFC and hippocampal system via thalamic nuclei, including the anterior thalamic nuclei. This system has been examined with spatial memory tasks (88), but little is known about the system during EBC. We suggest that the greater arousal state of schizophrenic patients may be due to impaired circuitry in the prefrontal–thalamic–hippocampal system, which is then less able to respond properly to stimuli that are behaviorally important.

## Effects of Neurotransmitters and Drugs on Eyeblink Conditioning

The EBC paradigm is an excellent model system to study behavioral pharmacology. Several drugs and transmitter systems have been examined using EBC. Acetylcholine (ACh) was one of the first neurotransmitters examined for effects on EBC. Given the involvement of the hippocampus in EBC (89), and the wide-spread role of ACh, Solomon et al. (90) examined the effects of scopolamine, a cholinergic, muscarinic antagonist on EBC in the rabbit. They found that systemically administered scopolamine severely impaired acquisition of delay EBC, but not when tested in rabbits that had their hippocampus ablated prior to the experiment. This demonstrates that a malfunctioning hippocampus (due to low ACh) is more of a detriment to learning than having no hippocampus at all, and suggests that abnormal neuronal transmission through the hippocampal system is likely to contribute to the cognitive impairments associated with schizophrenia.

Haloperidol was the next major drug examined for effects on EBC. This antipsychotic medication blocks dopamine (D2), alpha 1, and 5-HT2 (serotonin) receptors, among others, and has been shown to impair the acquisition rate for EBC (91). The impairment appeared to be due to an elevation in the threshold for an auditory CS to elicit CRs and suggests that the drug may be affecting attentional mechanisms and neuronal processing of the auditory cue since the effect was present when a 75 or 85-dB tone was used, but not when a 95-dB tone was used as a CS (92, 93).

The effects of serotonergic receptors on cognition, psychoses, and EBC deserve further review. An analysis of their effects on EBC has been investigated by John Harvey (94). He and his colleagues manipulated serotonergic receptors with agonists and antagonists during EBC and found that lysergic acid diethylamide (LSD) facilitated acquisition of CRs due to enhanced activation of the 2A/2C receptors unless the receptors were blocked by an antagonist, e.g., by Ritanserin (95). Since a 5HT1A agonist (8-OH-DPAT) had no effect, the effects of LSD are likely to be acting through the 2A/2C receptors rather than the 1A receptor. Harvey et al. (96) also increased the density of 5HT2A receptors in the frontal cortex by injecting MDL11,939 (a potent 5-HT2A antagonist) daily for 8 days prior to starting conditioning trials. The results indicated that the treated rabbits acquired CRs significantly faster than did rabbits given the vehicle control, and rabbits given the drug and explicitly unpaired stimuli exhibited <5% of trials with either spontaneous blinks or pseudo-CRs, suggesting that the drug was not acting on non-associative process.

Lastly, the *N*-methyl-d-aspartate (NMDA) receptor is the major excitatory receptor in the brain and is altered during learning and memory to facilitate ionic flow through its channel. Antagonists of the NMDA receptor (e.g., PCP, MK-801) are known to induce psychosis and have been found to impair EBC significantly in a dose-dependent manner in rabbits (77). Conversely, GLYX-13 (a novel NMDAR glycine-site functional partial agonist) facilitates acquisition of EBC in young and aging rats (27, 97).

## Other Behavioral Paradigms for Evaluating Schizophrenia

We have focused our discussion on EBC as a behavioral paradigm to evaluate the effects of the schizophrenic condition. This behavior could be considered as a conditioned avoidance response (CAR), the type of response that has classically been observed to evaluate the effectiveness of antipsychotic medications, i.e., suppression of the CAR (43). However, CAR paradigms typically evaluate responses that occur over the course of several seconds, as in moving away from a region to avoid a foot-shock. By contrast, movements related to EBC occur over the course of a fraction of 1 s. Regardless, both types of paradigms involve a CAR and should produce similar results. An examination of EBC under conditions that model the schizophrenic condition might allow a test of this hypothesis.

As alluded to earlier, EBC works so well with rabbits because it requires minimal behavioral output from the rabbit, and rabbits do not express much spontaneous behavior that might otherwise interfere with the behavior of interest. In terms of being able to use the rabbit to examine the neurobiology of schizophrenia in more detail, additional behaviors would be beneficial, both to add support to the results from EBC and to compare the rabbit to other established behavioral tests that are done in rodents. The novel object recognition (NOR) test is a popular test for declarative memory in rodents, especially for tests of schizophrenic-like impairments (44, 45, 98, 99). The test is done in two phases, an initial exploration phase where two identical objects are explored by the test animal, and a test phase that examines exploratory behavior after one of the objects has been replaced with a novel object after some period of time, e.g., 5–30 min. Rodents tend to favor the exploration of a novel object over the exploration of a familiar object, and the ratio of the time spent exploring one object relative to the other provides a cognitive index that can be evaluated.

The NOR paradigm has been used in rabbits by Hoffmann (100, 101) and was found to share similar properties with the rodent paradigm, i.e., the rabbits exhibited a preference for a novel object after a five minute delay (but not after a 20-min delay). Hoffman and colleagues also showed that acute administration of NMDA antagonists (ketamine and MK-801) significantly impaired NOR in the rabbits when the drug was administered 20 min before the sample phase of the test. The NOR paradigm in rabbits provides the opportunity to test the effects of the Meltzer paradigm for inducing schizophrenia by the chronic administration and subsequent washout of sub-anesthetic doses of NMDA receptor antagonists. Those results can then be compared directly with results from EBC studies to determine if the effects are generalized to multiple tests of memory and cognition, and if repeated doses of NMDA antagonists have prolonged effects. As noted above, the relative ease with which BOLD imaging studies can be done in rabbits offers the parallel opportunity to visualize the brain regions mediating the potential schizophrenia-like effect.

## Conclusion

Trace EBC is uniquely suited to examine cerebro-cerebellar interactions since the paradigm has been shown to require both the cerebellum and the forebrain. The additional requirement for awareness of the stimulus contingencies when a stimulus-free trace interval separates the two stimuli during a trial gives the paradigm good face validity. Although the paradigm has been used most often to study neural mechanisms mediating learning and memory in healthy adults, the paradigm can be used to detect impairments in neuropsychiatric diseases, especially schizophrenia. The paradigm is also quite translational in nature and animal models of schizophrenia can be examined with EBC in several species to allow an analysis from genes to molecules to behavior. The paradigm is frequently used in rabbits, rats, mice, and humans, but the rabbit model is particularly appealing given its tolerance for restraint and the ease of using it without the need for anesthetics or sedatives during functional imaging experiments. An animal model of schizophrenia is particularly suited to answer two important questions: (1) what therapeutics are best for treating both the cognitive and psychotic aspects of schizophrenia and (2) can neuroimaging reveal biomarkers of the disease and a determination of appropriate therapeutics? Forebrain-dependent trace EBC in the rabbit is positioned to answer these questions, and the relatively new demonstration of NOR in the rabbit (100) provides an additional test for cognitive impairments and amelioration of psychotic symptoms by antipsychotic drugs.

## Conflict of Interest Statement

The authors declare that the research was conducted in the absence of any commercial or financial relationships that could be construed as a potential conflict of interest.
